# Facile microfabrication of three dimensional-patterned micromixers using additive manufacturing technology

**DOI:** 10.1038/s41598-022-10356-z

**Published:** 2022-04-15

**Authors:** Doheon Koo, Hongyun So

**Affiliations:** 1 Department of Mechanical Engineering, Hanyang University, Seoul, 04763 South Korea; 2 Institute of Nano Science and Technology, Hanyang University, Seoul, 04763 South Korea

**Keywords:** Mechanical engineering, Surface patterning

## Abstract

This study investigates the manufacturing method of oblique patterns in microchannels and the effect of these patterns on mixing performance in microchannels. To fabricate three-dimensional (3D) and oblique patterns in microchannels, 3D printing and replica methods were utilized to mold patterns and microchannels, respectively. The angle and size of the patterns were controlled by the printing angle and resolution, respectively. The mixing efficiency was experimentally characterized, and the mixing principle was analyzed using computational fluid dynamics simulation. The analysis showed that the mixing channel cast from the mold printed with a printing angle of 30° and resolution of 300 μm exhibited the best mixing efficiency with a segregation index of approximately 0.05 at a Reynolds number of 5.4. This was because, as the patterns inside the microchannel were more oblique, “split” and “recombine” behaviors between two fluids were enhanced owing to the geometrical effect. This study supports the use of the 3D printing method to create unique patterns inside microchannels and improve the mixing performance of two laminar flows for various applications such as point-of-care diagnostics, lab-on-a-chip, and chemical synthesis.

## Introduction

Microfluidics is a fundamental principle that has been widely used for the establishment and commercialization of micro-total analysis systems (μ-TAS), lab-on-a-chip (LOC), and micro-electro-mechanical systems (MEMS)^1^. Moreover, it provided many tools such as mixers and micropumps that have advanced the knowledge base in many fields such as chemistry^2–4^, biology^5–7^, and mechanics^8–11^. Among the microfluidic devices, a micromixer, which functions as a microreactor, plays a key role in microfluidics because it can be used in many applications such as chemical analysis^12–14^, polymerization^15,16^, biological synthesis^17,18^ and point-of-care applications ^19^. However, it is difficult to fabricate a micromixer because it is not easy to mix two different fluids owing to the low Reynolds number which relies on diffusion^20–22^. Although various methods for manufacturing microfluidic devices exist, such as micromachining^23,24^ and laser ablation^25,26^, soft lithography based on polydimethylsiloxane (PDMS) is considered the most useful because of its facile fabrication and biocompatibility^27,28^. In particular, in the case of passive mixers, which do not require external forces, where the mixing channels have a complex geometric design for the splitting and recombination of the reacting fluid^29–31^, soft lithography has been considered an attractive method because of its accuracy and ability to print complex designs. However, the soft lithography method requires expensive equipment and photo-curable materials; moreover, a considerable amount of time is required to mask channel designs to produce microfluidic devices. Furthermore, the fabrication of channels with a three-dimensional (3D) profile using soft lithography is difficult and requires complex technology.

Accordingly, a technology for manufacturing microfluidic devices using 3D printing has recently emerged^32–35^. Compared to the existing methods, 3D printing is convenient for the fabrication of a 3D profile and has the advantage of a facile, rapid, and inexpensive fabrication process. In particular, fused deposition modeling (FDM)-type 3D printing has the advantage of rapid printing speed and inexpensive equipment; however, the fabricated channels have a rough surface and details may not be included owing to the low resolution of the printer. Recently, some studies on the fabrication of micromixers using FDM 3D printing have been conducted to overcome one disadvantage of FDM printing, which is the fabrication of channels with rough surfaces^36^. However, if the micromixers are manufactured with a 3D printer directly, the channel inside is not visible because of the printing resolution of the printer, which makes it difficult to perform and monitor many chemical experiments that require colorimetric observation. In addition, it is difficult to assess whether a channel is properly manufactured, although a transparent filament was used. Furthermore, the micromixer manufactured by this method comprises top and bottom channels with various surface patterns depending on the printing angle; however, the side walls of the channel have to unavoidably have surface patterns in one direction because of the characteristics of FDM 3D printing. This is because printing is achieved by stacking the filaments one on top of the other layer by layer. These patterns on the side walls disrupt the flow of fluid, inducing a decrease in the mixing efficiency.

**Figure 1:**
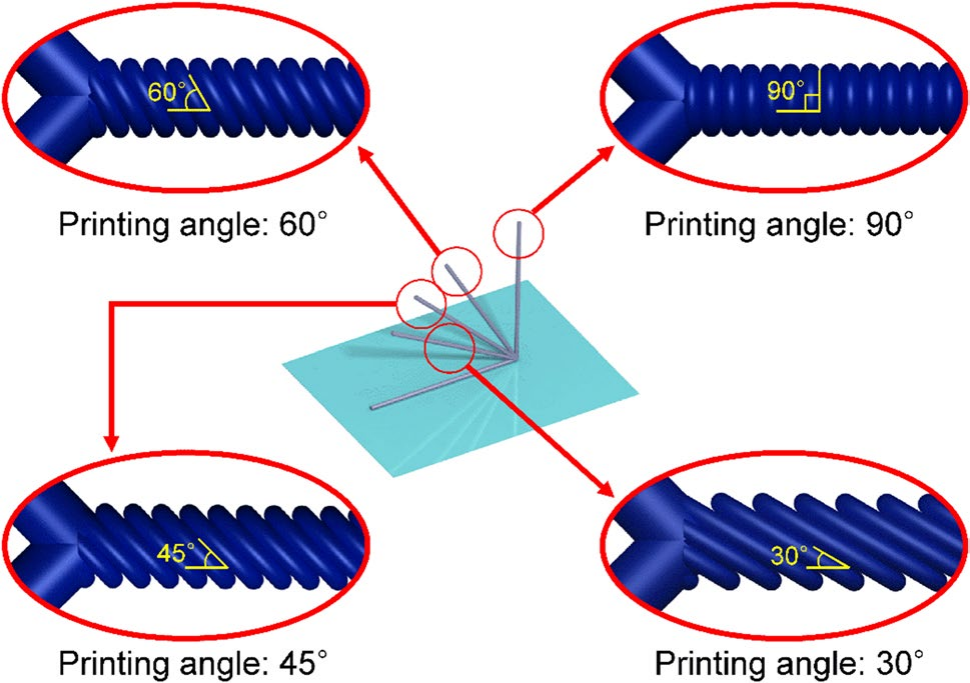
Schematic of the mixing channel molds printed by a 3D printer with various printing angles.

To address these engineering challenges, this study demonstrated an additive manufacturing method for a micromixer fabricated by the PDMS casting process using a 3D printing mold. The 3D printing mold has different printing angles (0, 30, 45, 60, and 90°), as shown in Fig. 1. In the CAD (computer-aided design) files, the mixing channel molds were designed as a continuous long cylinder; however, the actual printing products were similar to small discs stacked on top of each other. In addition, each mixing channel has a surface raster angle depending on the printing angle, which allows the fluid to flow in various forms under the influence of different viscous effects caused by the surface of the channel. Using this method, transparent channels with varied surface profiles can be achieved, and channels with 3D profiles can be easily fabricated without the use of expensive equipment and materials.

## Fabrication method

**Figure 2:**
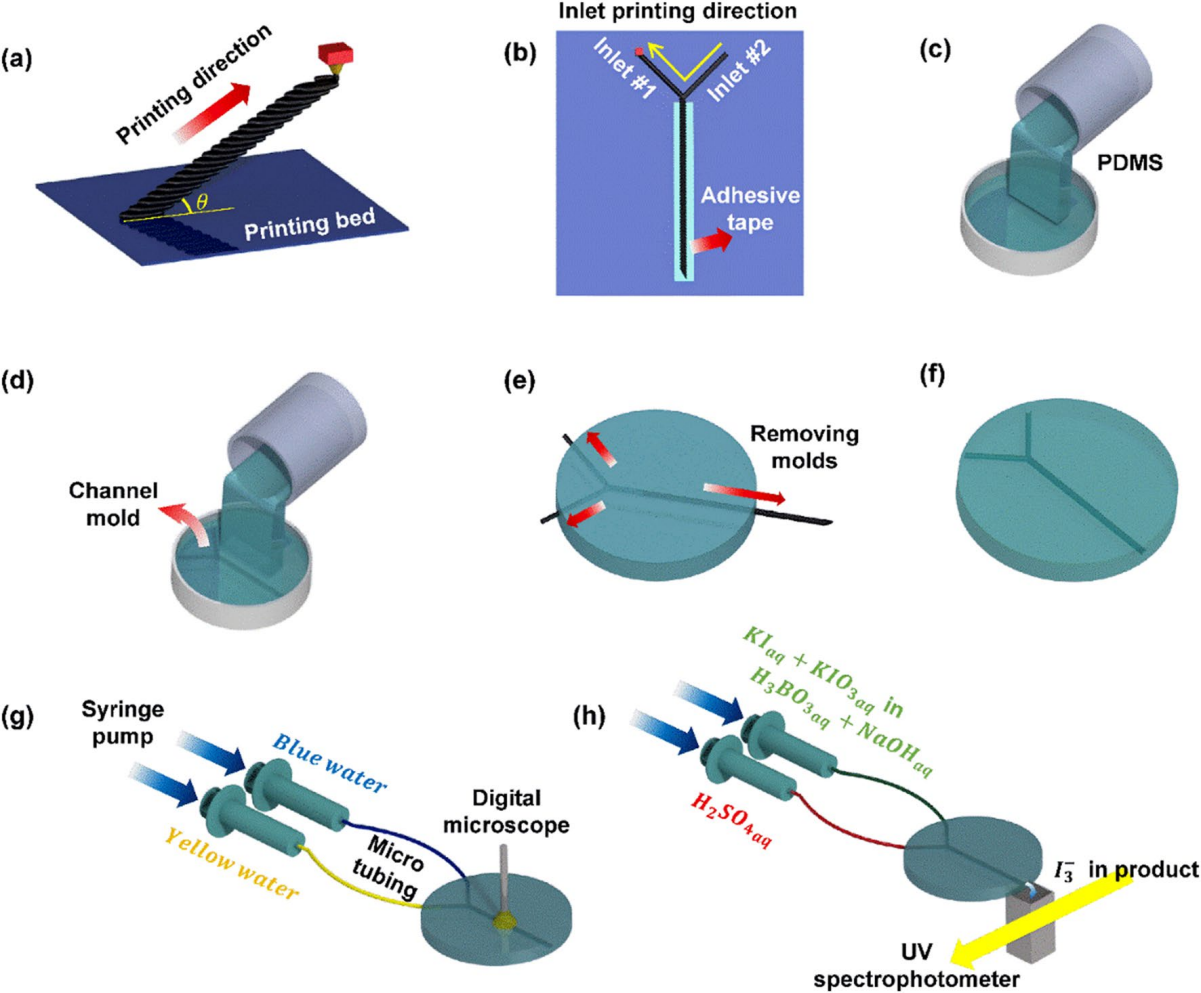
Fabrication and experimental process of a micromixer: (**a**) Printing mixing channel molds, (**b**) overlap printing for inlet channel, (**c**) first PDMS curing, (**d**) second PDMS curing, (**e**) removing each channel mold from cured PDMS molds, and (**f**) finalizing mixer channels. (**g**) Colorimetric experiment and (**h**) experiment for iodide–iodate competitive parallel reaction to investigate the mixing efficiency quantitatively.

The entire fabrication process of the micromixer is shown in Fig. 2a–f. First, the mixing channel molds were printed at various printing angles. The mixing channel and inlet channel molds were printed using an FDM 3D printer (Guider IIs, Flashforge 3D Technology Ltd.) and polylactic acid (PLA) filaments. Because PLA filaments have higher strength than acrylonitrile butadiene styrene and polyvinyl alcohol filaments, the molds can be printed stably without supporting structures, although the features of the mixing channel were oblique to the bed. In addition, the printing speed was set as fast as 100 mm/s such that deformation owing to the heat generated during printing can be minimized and the upper disc-shaped filament can press the one below immediately (Fig. 2a). The other printing settings are shown in Table 1. For the printing of the inlet channel, the printed main channel was attached to the bed with the raster surface facing up using an adhesive tape. The inlet channel was then overlap-printed with approximately 700 μm controlled by the predetermined printing direction of the inlet channel such that two inlets could be smoothly connected to the main channel (Fig. 2b). Through this process, the surfaces of the two inlet channels were smooth thus preventing pre-mixing before entering the main mixing channel. In addition to this, the joining point between the two inlet channels and the main mixing channel can be easily separated, which helps in the facile elimination of PLA channel molds from the cured PDMS mold. Subsequently, to create 3D patterns in the mixing channel, PDMS curing processes were performed twice for replicating all sides of the mixing channel, as shown in Fig. 2c,d. The first PDMS curing process was conducted without PLA channel molds at 70 °C for 3.5 h, and the second curing process was performed at 50 °C for 8 h, which was a temperature lower than the glass transition temperature of the PLA filament to prevent deformation during the curing process^37^. Because PDMS comprises the same curing agent ratio of 10:1 for the two-step curing processes, the cured PDMS molds could be perfectly combined as a bulk assembly without forming boundaries. Finally, after removing the PLA molds from the cured PDMS molds by hand (Fig. 2e) without chemical treatment^38^, the micromixer channel was realized, as shown in Fig. 2f. It was noteworthy that no physical damages or debris were observed even after pulling the PLA molds out of the cured PDMS molds. This might be due to the viscoelastic property of the cured PDMS, which can restore the 3D patterns against instant frictional forces.

**Table 1:** Printing conditions for PLA channel molds.

Printing speed (mm/s)	Nozzle travel speed (mm/s)	Nozzle diameter (mm)	Nozzle temperature (°C)	Bed temperature (°C)	Printing resolution (μm)
100	100	0.4	230	50	200, 300

## Experimental section

### Measurement of mold parameters

To investigate the mold morphology created using FDM 3D printing, a digital microscope (UM12, ViTiny, Microlinks Technology Corp.) was used. The images of the mixing channel molds were taken with the raster pattern facing up to observe the width of the channel and surface, which varied depending on the printing angles. The captured images were then processed using the ImageJ program to measure the channel width. A vernier caliper (CD-15APX, Resolution of 0.01 mm, Mitutoyo Corp.) was used to measure the channel height.

In this study, Reynolds numbers ($Re_{L}$) were calculated by Eq. (1)^39^ to classify the flow characteristics for each channel,

1 $$Re_{L} = \frac{\rho uL}{\mu }$$

where $u$ is the velocity of the fluid and $L$ is the characteristic length. The density ($\rho$) and dynamic viscosity ($\mu$) were calculated using the interpolation equation of water^39^,

2 $${\rho } \approx 1000 - 0.0178\left[ {{\text{T}} - 4} \right]^{1.7} \pm 0.2\%$$

3 $${\text{ln}}\frac{\mu }{{\mu_{0} }} \approx - 1.704 - 5.306 \times z + 7.003 \times z^{2}$$

where T [°C] is temperature of water, ${\text{z}} = \frac{273}{{T + 273}}$ is temperature ratio and *μ*_0_ = 1.788 × 10^−3^ N s/m^2^ is dynamic viscosity of water at 0 °C . The density and dynamic viscosity at 20 °C, which is the environmental temperature of our experiments, were calculated as 998 kg/m^3^ and 1.003 × 10^−3^ N·s/m^2^, respectively. In order to obtain *u* and *L* in Eq. (1), following equations were used,

4 $$u = \frac{Q}{A}$$

5 $$L = \frac{4A}{P}$$

where *Q* is the flow rate provided by a syringe pump, $A$ is the cross-sectional area, and $P$ is the wetted perimeter. When calculate the $A$ and $P$, the cross-sectional shape of the channel was simplified as an ellipse. Through the measured values of channel width as major radius and channel height as minor radius of the ellipse, the cross-sectional area and characteristic length were obtained using the following equations,

6 $$A = {\uppi }ab$$

7 $$P = 4\mathop \smallint \limits_{0}^{\pi /2} \sqrt {a^{2} sin^{2} \theta + b^{2} cos^{2} \theta } d\theta$$

where *a* and *b* is major radius and minor radius of cross-section of channel, respectively.

### Flow analysis in the mixing channel

The flow in the mixing channel was visually observed through colorimetric experiments using deionized (DI) water of two different colors. One inlet was injected with blue food dye (Royal Blue Icing Color, Wilton) and the other was injected with yellow food dye (Lemon Yellow Icing Color, Wilton). The dyed DI water was injected using syringes pushed by a syringe pump (PHD ULTRA, Harvard Apparatus Ltd.). The syringes and manufactured micromixer were connected by polyetheretherketone tubing (Tub PEEK 1569, IDEX Health & Science), as shown in Fig. 2g, and the connection between the tubing and the micromixer was coated with epoxy to prevent leakage of fluid. The colorimetric experiment was repeated using five channels with the same channel geometry, and measurement was conducted three times per each channel under different flow rates (60, 120, 240, 480, and 960 μL/min). Finally, COMSOL Multiphysics software was used to analyze the cross-sectional flow in the mixing channel and investigate the mixing principles. The simulation details are provided in Supplementary Material.

### Quantification of the performance of the micromixer

To quantify the performance of the micromixer, the iodide–iodate competitive parallel reaction system, also called the Villermaux–Dushman method, was used^40^. For this experiment, reagents comprising sodium hydroxide (NaOH, Duksan Pure Chemicals), boric acid (H_3_BO_3_, Kanto Chemical Corp.), potassium iodide (KI, Samchun Pure Chemical), potassium iodate (KIO_3_, Jensei Chemical Corp.), and sulfuric acid (H_2_SO_4_, Deajung Chemicals & Metals Corp.) were prepared, and the products were measured using a UV–visible spectrophotometer (Genesys 180, Thermo Fisher Scientific), as shown in Fig. 2h. The overall experimental method was performed based on a method suggested in previous studies^41,42^. The concentration of each reagent was 0.25, 0.5, 0.035, 0.007, and 0.015 mol/L for NaOH, H_3_BO_3_, KI, KIO_3_, and H_2_SO_4_, respectively. In this study, the concentrations of H_3_BO_3_ and NaOH were larger than those of other reagents to prevent dilution of the product for accurate measurement. This manipulation is reasonable because the segregation index, which represents the mixing performance, can be expressed as the ratio of products to total reagents. The segregation index ($X_{s}$) is defined as follows^42^:

8 $$X_{s} = \frac{Y}{{Y_{ST} }}$$

where *Y* is the ratio of the iodide–iodate reaction with acid following reaction (ii), $Y_{ST}$ is the value of *Y* in an ideal segregation case when the micro mixing process is infinitely slow. $Y$ and $Y_{ST}$ are expressed as follows^42^:

9 $${\text{Y}} = { }\frac{{2\left( {M_{{I_{2} }} + M_{{I_{3}^{ - } }} } \right)}}{{M_{{H_{0}^{ + } }} }} = \frac{{2\left( {C_{{I_{2} }} + C_{{I_{3}^{ - } }} } \right)\left( {Q_{1} + Q_{2} } \right)}}{{C_{{H_{0}^{ + } }} Q_{2} }}$$

10 $$Y_{ST} = \frac{{6C_{{IO_{3}^{ - } }} }}{{6C_{{IO_{3}^{ - } }} + C_{{H_{2} BO_{3}^{ - } }} }}$$

where $M$ and $C$ are the molar number and concentration of subscript ions, respectively. $Q_{1}$ and $Q_{2}$ are the flow rate through two inlet channels. The neutralization of dihydroborate ions is as follows^41^:

i $${\text{H}}_{2} {\text{BO}}_{3}^{ - } + {\text{ H}}^{ + } { } \leftrightarrow {\text{H}}_{3} {\text{BO}}_{3} ,\;{\text{quasi}} - {\text{instantaneous}}$$

The iodide-iodate competitive parallel reactions are as follows^41^:

ii $$5{\text{I}}^{ - } + {\text{IO}}_{3}^{ - } + 6{\text{H}}^{ + } { } \leftrightarrow { }3{\text{I}}_{2} + 3{\text{H}}_{2} {\text{O,}}\;{\text{very fast}}$$

The reaction wherein iodide ions yield triiodide ions for quasi-instantaneous equilibrium is as follows^41^:

iii $${\text{I}}_{2} + {\text{ I}}^{ - } { } \leftrightarrow {\text{ I}}_{3}^{ - } ,\;{\text{quasi}} - {\text{instantaneous}}$$

To calculate $X_{s}$, concentrations of triiodide ions ($C_{{I_{3}^{ - } }}$) and iodine ($C_{{I_{2} }}$) as a product were derived in different ways. Triiodide ions were measured using a UV–visible spectrophotometer with peaks at 288 and 352 nm, and calculated using the Beer–Lambert law^41^ with the measured absorbance (*D*) as follows:

11 $$C_{{I_{3}^{ - } }} = \frac{D}{\varepsilon l}$$

where $l$ is the optical path length and ${\upvarepsilon }$ is the molar extinction coefficient of $I_{3}^{ - }$ at 352 nm. The concentration of iodine was calculated by the mass balance on the concentration of iodine as follows^42^:

12 $$C_{{I^{ - } }} = \frac{1}{2}C_{{\left( {I^{ - } } \right)_{0} }} - \frac{5}{3}C_{{I_{2} }} - \frac{8}{3}C_{{I_{3}^{ - } }}$$

13 $$K_{B} = \frac{{C_{{I_{3}^{ - } }} }}{{C_{{I_{2} }} C_{{I^{ - } }} }}$$

where $C_{{\left( {I^{ - } } \right)_{0} }}$ is the initial concentration of $I^{ - }$ ions in the solution before mixing, $K_{B}$ is the equilibrium constant of reaction (iii), which is a function of temperature (${\text{T}}$ [K])^43^.

14 $${\text{log}}_{10} K_{B} = \frac{555}{T} + 7.355 - 2.575{\text{log}}_{10} T$$

In the experiment to measure mixing efficiency using the iodide-iodate competitive parallel reaction system, four channels for each design were measured three times, and a total 12 segregation index values were averaged and compared.

## Results and discussion

**Figure 3:**
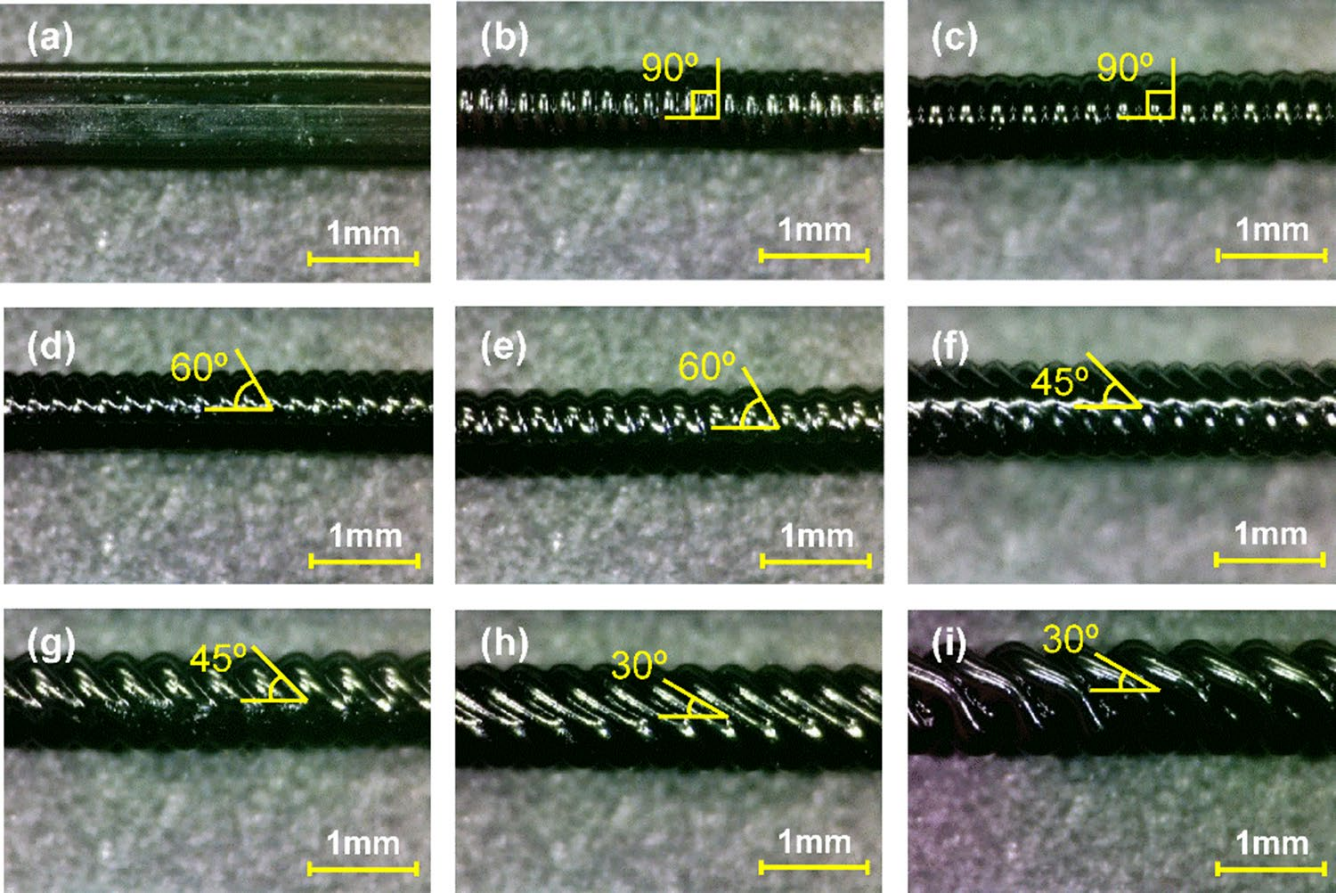
Optical images of the PLA mixing channel molds with various printing (raster) angles and resolutions: (**a**) 0°–200 μm, (**b**) 90°–200 μm, (**c**) 90°–300 μm, (**d**) 60°–200 μm, (**e**) 60°–300 μm, (**f**) 45°–200 μm, (**g**) 45°–300 μm, (**h**) 30°–200 μm, and (**i**) 30°–300 μm.

Figure 3 shows optical images of the mixing channel molds printed by an FDM 3D printer. Because the direct printing of mixing channels using a 3D printer is inefficient and unstable as the channel length increases, the replica (casting) method using mixing channel molds was utilized in this study to achieve the desired channel design^44,45^. Although some printing instabilities occurred during the direct printing of PLA channel molds, the PDMS channel cast from the single PLA channel mold exhibited almost uniform shape and dimensions including channel width, pattern angle, and pattern thickness. In addition, all the inner surfaces of the mixing channel can have rough patterns using the replica method, which is not achievable with conventional direct printing methods. To investigate the effect of the raster (printing) angle on the mixing behavior and efficiency, a mixing channel mold without surface patterns was also prepared with a printing angle of 0°, as shown in Fig. 3a. The four different printing angles were set to 30, 45, 60, and 90°, and two different printing resolutions of 200 and 300 μm were utilized, as shown in Fig. 3. As the printing resolution increased from 200 to 300 μm, larger patterns were generated, and the cross-sectional area of the mixing channel increased simultaneously. It was also noticeable that the angle of inclination of each pattern was almost identical to the printing (raster) angle (see Fig. 1), leading to the facile design of inclined groove patterns in micro mixing channels.

**Figure 4:**
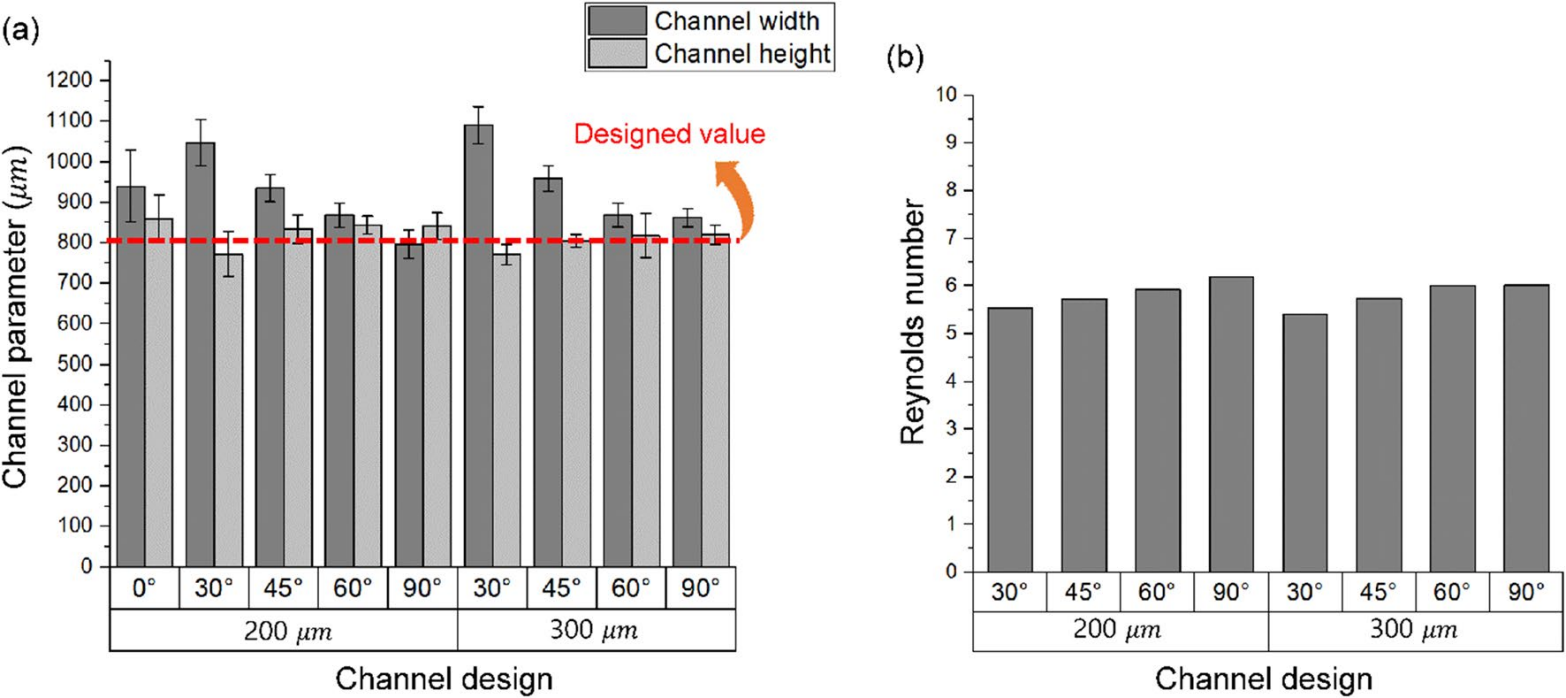
Characteristic of the fabricated mixing channel: (**a**) dimensions of mixing channel molds, (**b**) Reynolds number of the mixing channel with a flow rate of 120 μL/min at each inlet.

The measured dimensions (width and height) of the printed channel molds are shown in Fig. 4a. To evaluate the precision and accuracy of manufacturing technique, five different molds for each channel design were used, and the width and height at 10 different locations per each mold were measured. In the CAD file of the 3D printing model, a cylinder with a diameter of 800 μm was designed; however, a relative difference existed in the channel width along the channel height in the printed molds. This phenomenon occurred because the more oblique printing angle and the higher resolution had a smaller area where the printed upper disc shapes were supported. Consequently, the difference in channel width between the designed and manufactured dimensions becomes larger as the printing angle decreases from 90 to 30° and the printing resolution decreases from 200 to 300 μm. Although the dimensions of the mixing channels slightly differed, the Reynolds number calculated by considering the cross-sectional area of each mixing channel showed a similar range between 5.4 and 6.1 under a flow rate of 120 μL/min at the inlet, as shown in Fig. 4b. Therefore, the change in flow behavior and mixing efficiency owing to the channel dimensions were relatively negligible in this study.

**Figure 5:**
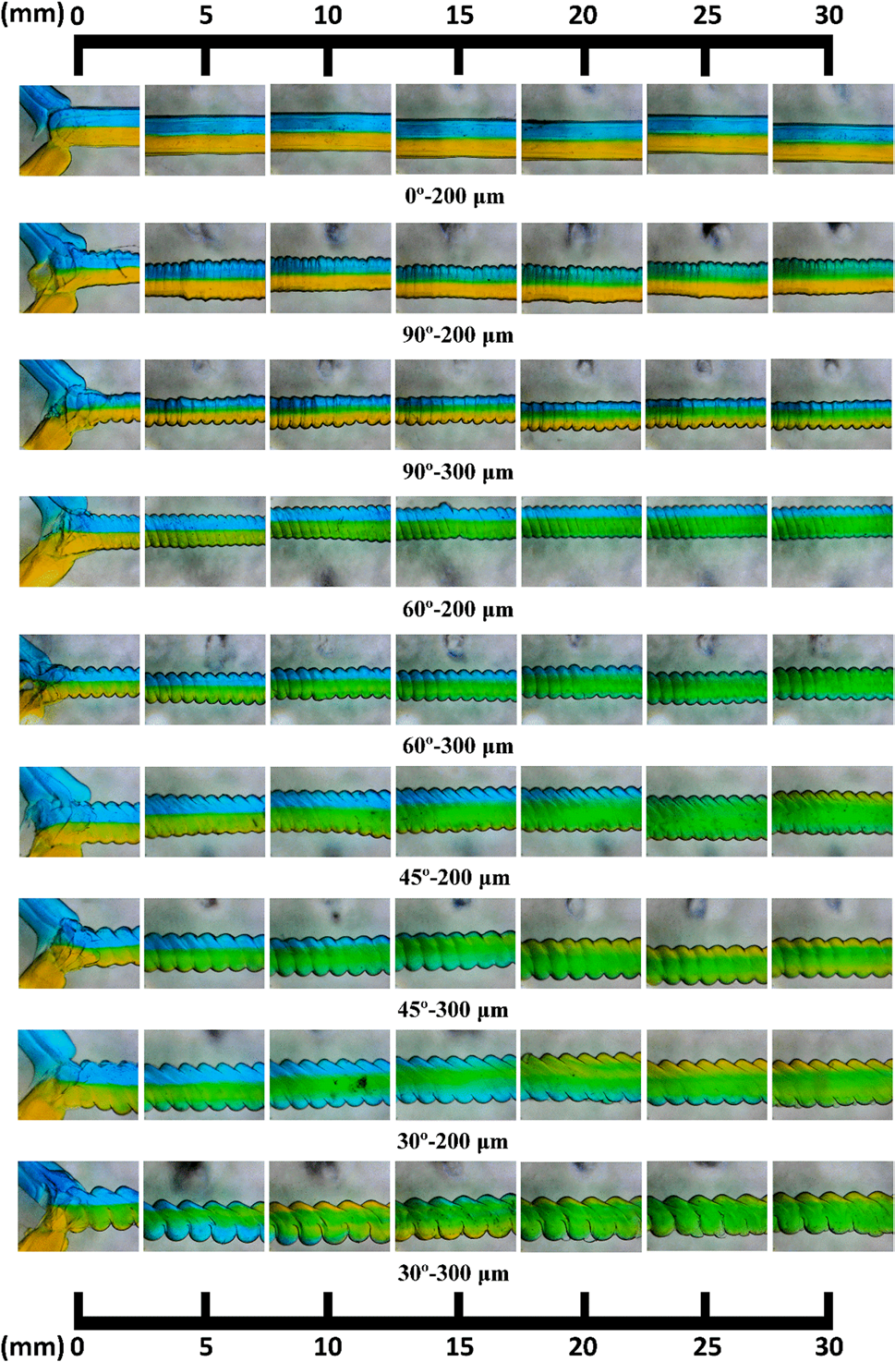
Optical images of the colorimetric experiments with a flow rate of 120 μL/min at each inlet. The water dyed in blue and yellow colors was injected at the two inlets. The green color indicated the fluid mixed with blue and yellow-colored water.

To analyze the fluid behavior in the mixing channel, colorimetric experiments were conducted using water with color (blue and yellow) dyes; the results are shown in Fig. 5. The applied flow rate for each inlet was set to 120 μL/min, and the total length of all the mixing channels was 35 mm. As expected, the two fluids were not mixed in the mixing channel printed with a printing angle of 0° (i.e., straight channel without surface patterns). However, the two fluids exhibited distinct behaviors as the surface patterns varied in the mixing channels. For the mixing channel cast from the mold printed with an angle of 90° (i.e., channel marked as “90°–200 μm” and “90°–300 μm” in Fig. 5), two fluids flowed in a straight flow direction. The green area, which indicates the fluid containing mixed blue and yellow water, was thicker in the case of “90°–300 μm” than in the case of “90°–200 μm”, indicating that the surface pattern affected the mixing behavior in the micromixer. Although the green area appeared slightly at the end of the mixing channel, a large portion of unmixed blue and yellow water still existed in the mixing channel, resulting from insufficient mixing performance in mixing channels with 90°-patterns. In contrast, it was observed that the blue water started penetrating the flow of yellow water at the raster (pattern) angle of 60° (see “60°–200 μm” and “60°–300 μm” cases in Fig. 5). Furthermore, as the raster angle of patterns and printing resolution (i.e., pattern thickness) were more oblique and thicker, the mixing of the two fluids became stronger. It should be noted that the position where the two fluids started mixing, resulting in green color, gradually approached the inlet location (see the case of “30°–300 μm”). This implies that the two fluids immediately start mixing in the mixing channel when the angles of the surface pattern and thickness were 30° and 300 μm, respectively. Although the different quality of the joining part could cause the early or delayed mixing, the inclined angle and thickness of the surface pattern might significantly attribute to the mixing behavior rather than the quality of the joining part.

**Figure 6:**
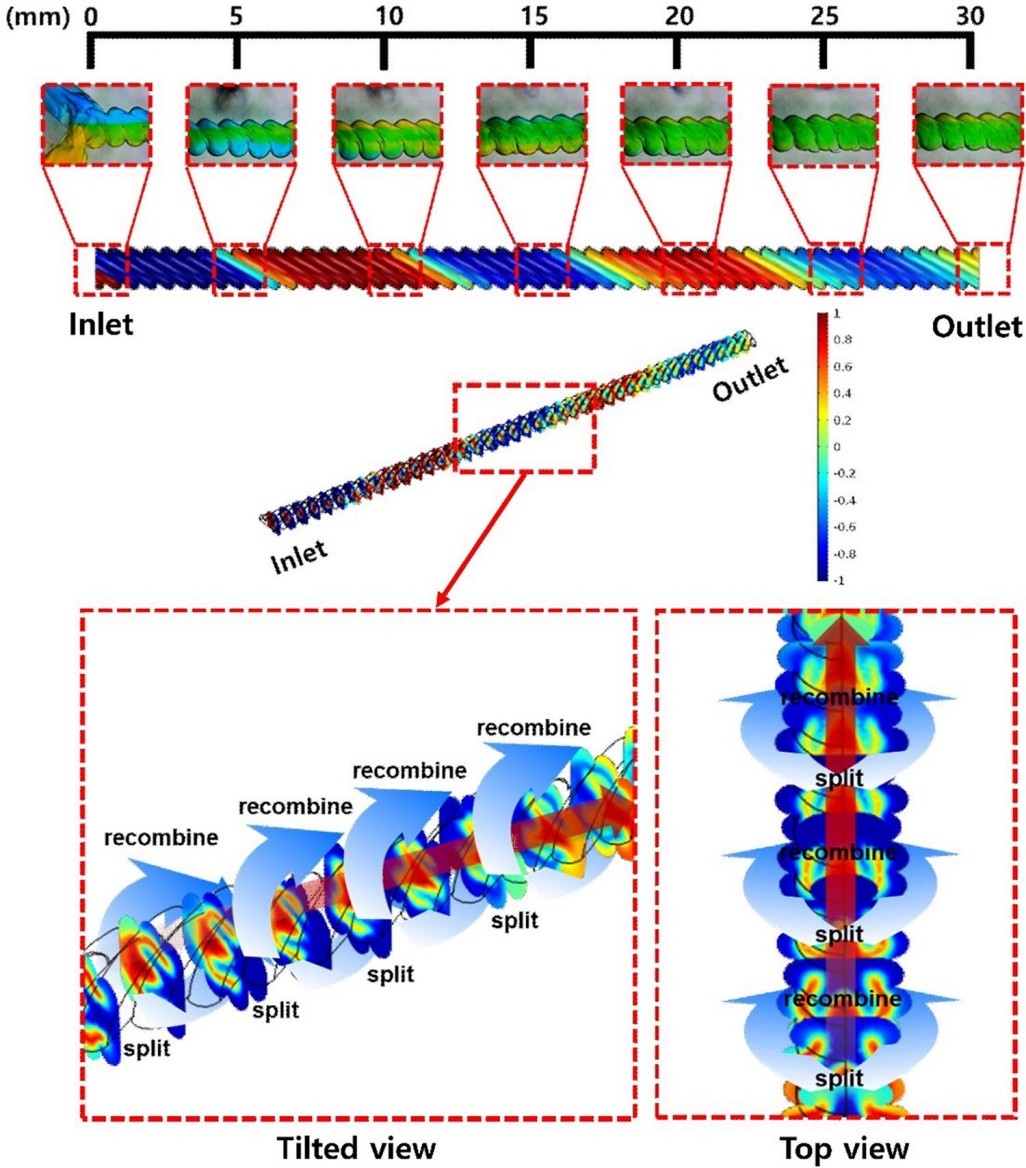
Mixing behavior of the fluids in the mixing channel estimated by CFD. “Split” and “recombine” as one fluid surrounds the other. These alternative “split” and “recombine” actions caused by the raster angle of the channel helped in the mixing of the two fluids.

Computational fluid dynamics (CFD) was performed to investigate in greater depth the flow behavior and mixing principle in the mixing channel. The CAD modeling for CFD analysis was redesigned using the measured dimensional parameters shown in Fig. 4a, as indicated in Fig. S1 in Supplementary Material. The shape of mesh, mesh grid dependency, and simulation results are shown in Table S1 and Figs. S2–S5 in Supplementary Material. Figure 6 shows the comparison between the simulation and the colorimetric experiment in the mixing channel of “30°–300 μm”. The flow color indicates the concentration of the fluid. The red and blue colors correspond to a concentration of 1 and − 1, respectively, and change to a green color (concentration of 0) as they mixed properly. In simulation results, the fluid in direct contact with the surface of the mixing channel flowed along the raster angle of the surface. The raster angles of the mixing channel are in the same direction as the top and bottom of the channel because the discs are printed layer by layer when the mold of the mixing channel is printed owing to the characteristics of the FDM 3D printing method. This surface pattern differs from the helical shape, where the raster patterns on the top and bottom surfaces are reversed. With these surface patterns, one fluid flows as it is divided through the top and bottom surface raster patterns at one point on the side of the mixing channel and the other fluid flows between them. Owing to this unique surface pattern, one fluid is surrounded by the other fluid in the mixing channel, and this process is alternatively repeated along the mixing channel. Therefore, the two fluids experienced “split” and “recombine” behavior from the viewpoint of mixing. This mixing principle was further demonstrated in another experiment.

**Figure 7:**
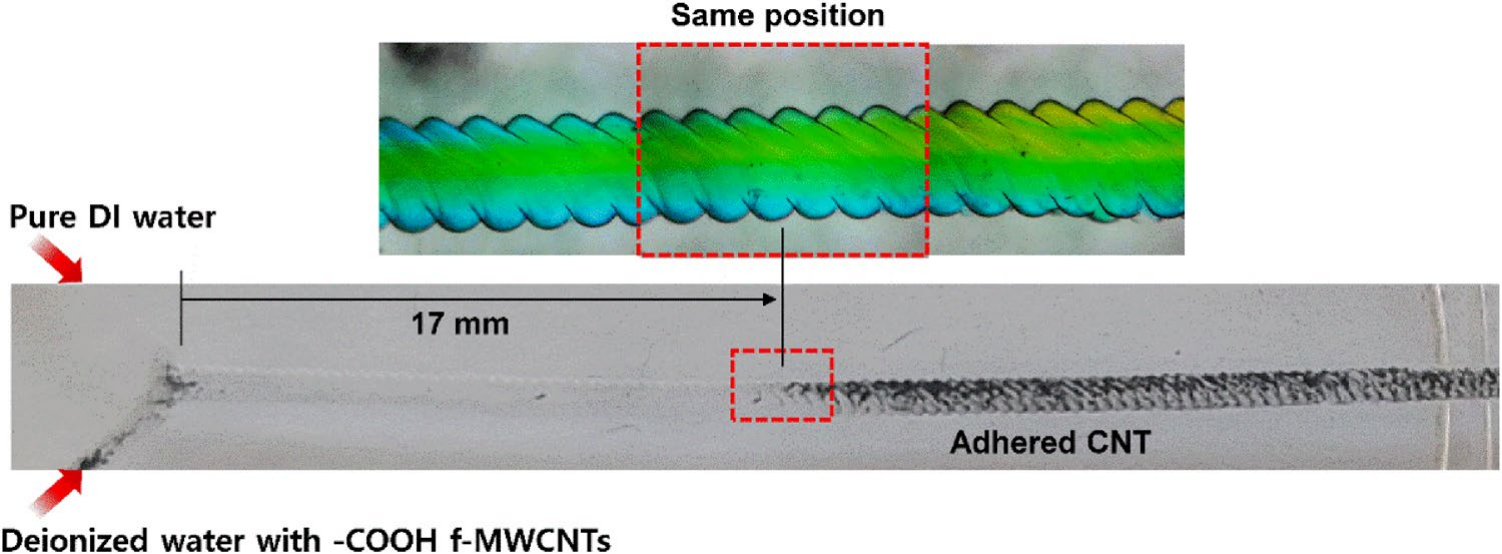
Result of the experiment using CNTs-dispersed water to verify the “split” and “recombine” behavior of fluids in the mixing channel. The flow rate for both inlets was 120 μL/min.

Figure 7 shows the mixing experiment to verify the “split” and “recombine” actions during the mixing process in the mixing channel of “30°–200 μm”. For this experiment, pure DI water was injected into the first inlet, and a carboxyl-functionalized multi-wall carbon nanotube (–COOH f-MWCNTs, OD: 20–30 nm, US Research Nanomaterials Inc.) dispersed in DI water was injected into the second inlet. Because the CNTs well adhere to the PDMS surface, the flow along the raster pattern of the mixing channel during the “split” and “recombine” actions can be simply verified. As shown in Fig. 7, at the beginning of the mixing channel, the water where the CNTs were dispersed was surrounded by pure DI water injected from the first inlet; thus, the CNTs could not adhere to the channel surface. As the mixture flowed downward, the CNT-dispersed water began splitting at approximately 17 mm away from the inlets; then CNTs gradually adhered to the inner surface of the channel. At this position, one fluid flowed while surrounding the other fluid, similar to the experimental result using blue- and yellow-colored water (see inset image in Fig. 7) and simulation results in Fig. 6.

**Figure 8:**
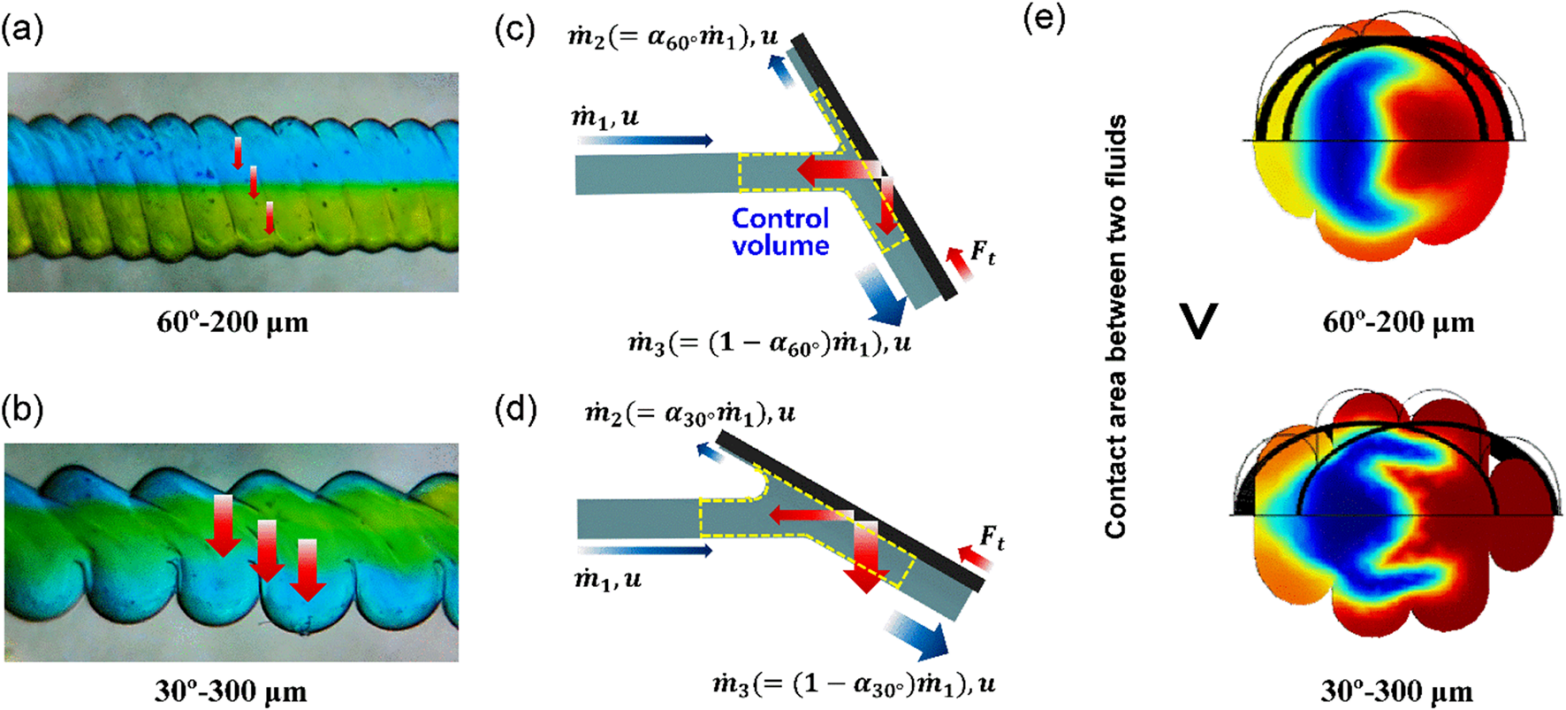
Fluidic analysis in the mixing channel from a fluid dynamic point of view: (**a**) flow in the 60°–200 μm, (**b**) schematic of control volume analysis of force balance with the 60°-oblique pattern, (**c**) flow in the 30°–300 μm, and (**d**) schematic of control volume analysis of force balance with the 30°-oblique pattern. (**e**) Cross-sectional view of two fluids, and comparison of interfacial shape during “split” and “recombine” actions with respect to the angle of patterns.

The “split” and “recombine” behavior of flow became more distinct as the printing angle decreased from 90 to 30° and the printing resolution decreased from 200 to 300 μm. Figure 8 depicts the cause of this phenomenon using an example of a simple fluid dynamic problem^39^. From the point of view of a fluid flowing along the inner surface of the mixing channel, the pattern on the surface can be considered as an obstacle in the path of the fluid flow. In the channels with 90°-patterns, these patterns can be considered as obstacles aligned perpendicular to the direction of the fluid flow. When this flow is analyzed using the mass continuity and momentum conservation equations through control volume analysis, the same portion of fluid flows upward and downward after colliding with the 90°-patterns because no reaction force exists in the direction perpendicular to the flow. Therefore, the fluid could not be mixed efficiently. By contrast, for mixing channels with inclined patterns (e.g., 60°-pattern), the different flow rates are separated upward and downward because of the different reaction forces perpendicular to the flow caused by the inclined obstacle (pattern), as shown in Fig. 8a,b. The force balance calculated through the control volume analysis was as follows:

15 $$\sum F_{t} = 0 = \dot{m}_{2} u + \dot{m}_{3} \left( { - u} \right) - \dot{m}_{1} u{\text{cos}}\left( {\theta + \pi } \right) = \left( {\alpha - \left( {1 - \alpha } \right) + {\text{cos}}\theta } \right)\dot{m}_{1}$$

where α is the ratio of the flow separated by the inclined pattern and is calculated as α = (1−cosθ)/2 from Eq. (15). This result implies that the ratio of the flow rate separating up and down by the obstacle is affected only by the inclined angle of the pattern, regardless of the flow rate. Therefore, a more inclined pattern induces the fluid to flow better along the pattern, as shown in Fig. 8c,d. This was further demonstrated using a colorimetric experiment and CFD simulation with different flow rates, indicating the significant effect of the inclined angle of the pattern compared to the flow rate, as shown in Fig. S5 in Supplementary Material. In the case in which the location where the blue and yellow water were reversed was closer to the inlet of the channel as the angle of the pattern decreased from 90 to 30°, the analytical results from Eq. (15) reasonably explained the effect of the inclined pattern angles on the mixing performance. This phenomenon also occurs when the printing resolution lowered from 200 to 300 μm. As shown in Fig. 5, as the printing resolution changed from 200 to 300 μm, the thickness of the surface pattern became thicker and rougher. These geometries increase the amount of fluid flowing along the surface pattern to promote transverse flow in the longitudinal direction of the channel. In addition, the shape of the interface between two fluids during the “split” and “recombine” actions can be varied based on the channel shape. Figure 8e shows the cross-sectional view of the mixing channels of “30°–300 μm” and “60°–200 μm”. It was noticeable that the interface area between the two fluids (i.e., blue- and red-colored water) was wider in the case of “30°–300 μm” (approximately 30% larger) than “60°–200 μm” owing to the geometrically oblique patterns. This implies that the wider contact interface accelerated the diffusion between the two fluids, based on Fick’s first law of diffusion^46^.

**Figure 9:**
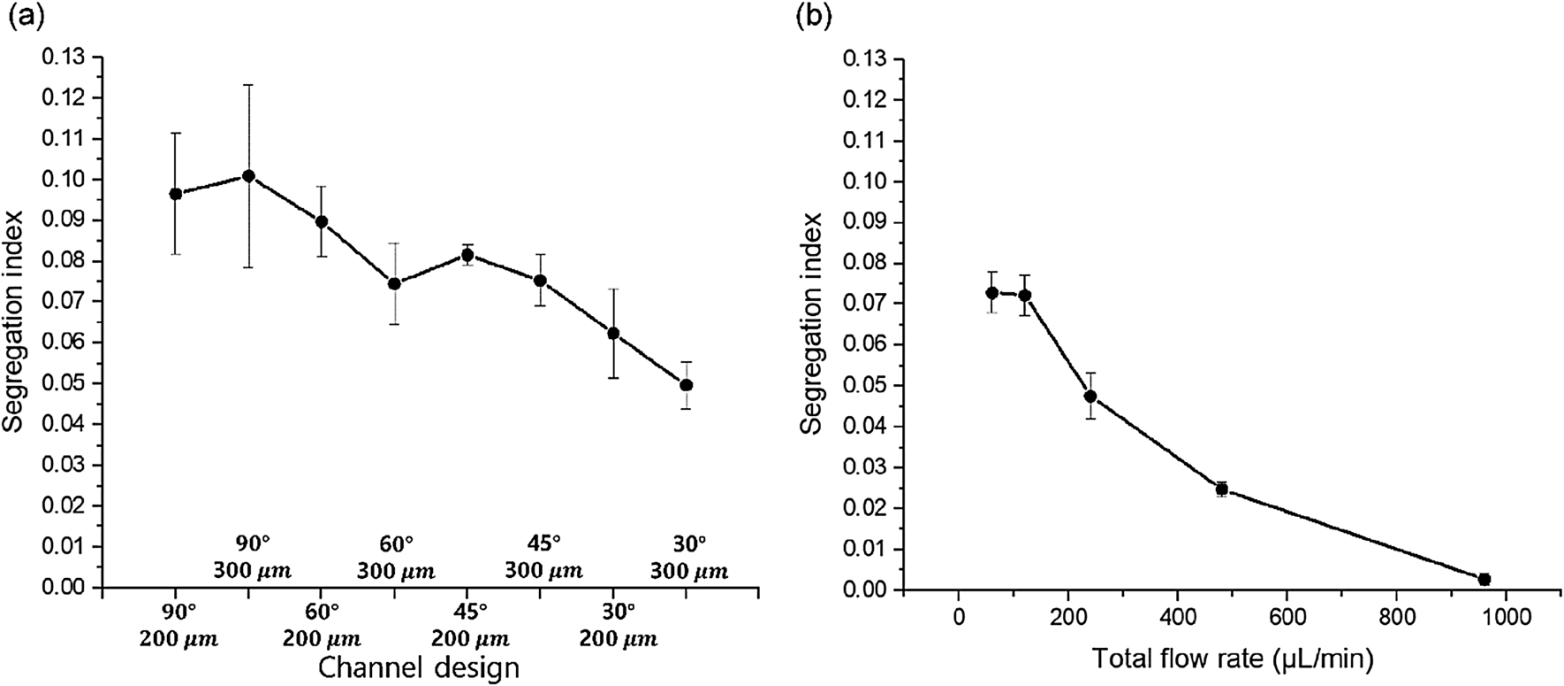
Segregation index calculated with respect to (**a**) channel design (printing angle and resolution) at a flow rate of 240 μL/min and (**b**) total flow rate in the mixing channel of “30°–300 μm”.

Iodide–iodate competitive parallel reactions were used to quantitatively characterize the mixing performance. The mixing performance can be approximately estimated by comparing the green area (i.e., blue and yellow water mixed) in the colorimetric experiment, as shown in Fig. 5. However, this qualitative method cannot provide an accurate value for the quantification of the mixing efficiency in a 3D-shaped mixer. Figure 9a shows the segregation index with respect to the mixing channel designs. For the mixing channels cast from the mold printed with a printing angle of 90° (i.e., “90°–200 μm” and “90°–300 μm”), a segregation index of approximately 0.1 was obtained, and this value was used as a criterion for insufficient mixing. As the inclined angle of the patterns decreased from 90 to 30°, the segregation index decreased owing to the “split” and “recombine” actions, which helped the two fluids mix sufficiently. In particular, the mixing channel cast from the mold printed with a printing angle of 30° and printing resolution of 300 μm (i.e., “30°–300 μm”) exhibited the lowest segregation index (i.e., the highest mixing efficiency). In addition, the mixing channel of “30°–300 μm” showed reasonably good mixing performance, which was comparable to those of previous micromixers at the low Reynolds number flows between 1.35 and 21.6^47–49^, as shown in Fig. 9b. It was noticeable that the mixing efficiency was enhanced as the flow rate in the mixing channel increased; the same tendency as in previous studies using the iodide–iodate competition parallel reaction^40,42,47–51^. Therefore, two different fluids can be efficiently mixed using a micromixer fabricated by replica and 3D printing manufacturing.

## Conclusion

This study demonstrated the manufacturing of PDMS-based micromixers using the printed mold and replica (casting) method. The micromixer made by this method included mixing channels with various surface shapes depending on the printing angle and resolution. Through the colorimetric experiment, it was observed that the oblique patterns were helpful for efficient mixing compared to the plain channel and fabricated channel with vertically aligned patterns (i.e., 90°-patterns). As the printing angle decreased from 90 to 30°, two fluids rapidly mixed owing to the “split” and “recombine” behaviors in the mixing channel, which was confirmed using CFD simulation. To characterize the mixing efficiency quantitatively, an iodide–iodate competitive parallel reaction was performed, and the segregation index was calculated. Consequently, the mixing channel cast from the mold printed with a printing angle of 30° and resolution of 300 μm exhibited the lowest segregation index, indicating the highest mixing efficiency compared to other types of mixing channels. This study supports the use of 3D printing methods to manufacture facile and inexpensive microfluidic devices for various engineering applications such as point-of-care diagnostics, lab-on-a-chip, and chemical synthesis.

## Supplementary Information

Supplementary Information.

## Data Availability

The data that support the findings of this study are available from the corresponding author upon reasonable request.
