# Supplementary material for: Facile microfabrication of three dimensional-patterned micromixers using additive manufacturing technology
Source: Sci Rep. 2022 Apr 15;12:6346. doi: 10.1038/s41598-022-10356-z (PMC9012767; doi:10.1038/s41598-022-10356-z)
Supplement: Supplementary file 1 — Supplementary Information. [file 41598_2022_10356_MOESM1_ESM.pdf]

Supplementary Material

Facile Microfabrication of Three Dimensional-Patterned Micromixers using Additive Manufacturing Technology

Doheon Koo [1] and Hongyun So[1,2*]

[1]Department of Mechanical Engineering, Hanyang University, Seoul 04763, South Korea
[2]Institute of Nano Science and Technology, Hanyang University, Seoul 04763, South Korea
[*]Corresponding Author. E-mail : hyso@hanyang.ac.kr

I. CFD simulation: design and boundary conditions

A CFD simulation was performed to analyze the flow in the mixing channel. The CAD modeling for the CFD simulation was redesigned with the measured width and height of the fabricated mixing channel for simulation close to reality. For example, discs with a height of 200 μm and 300 μm are vertically stacked according to the printing resolution in the 90º-pattern design. The cross-section of these disks is composed of an ellipse where the major axis is the length of the width and the length of the short axis is the length of the height. However, in the case of mixing channels printed obliquely, the major length of the cross-section of the disks becomes the value multiplied by 1/sinθ in the width as the printing angle (θ), and the disks are stacked while being shifted by the value multiplied by cosθ in the height of the disk, as shown in Fig. S1. In addition, the computation domain was reduced by cutting the overall domain in the direction of symmetry along the channel to obtain more accurate simulation results by creating more mesh elements within the limited memory, as shown in Figs. S2(a) and S2(b).

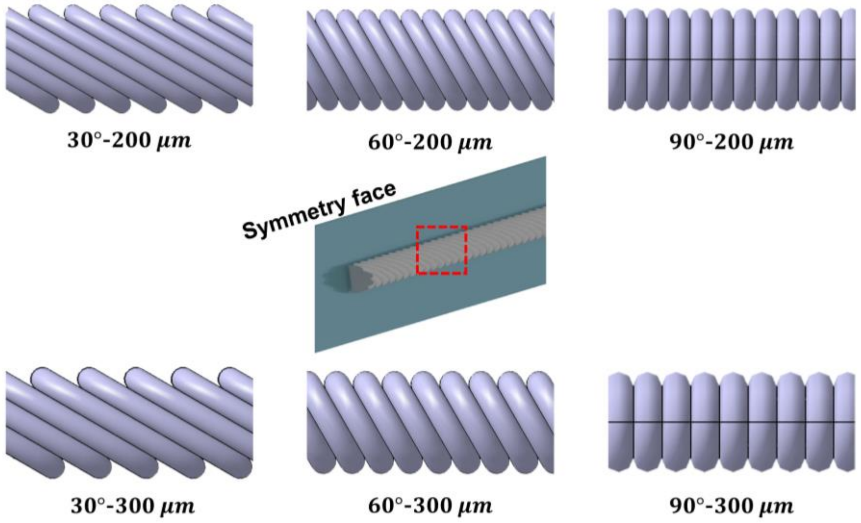

Fig. S1 CAD design of each mixing channel for CFD simulation.

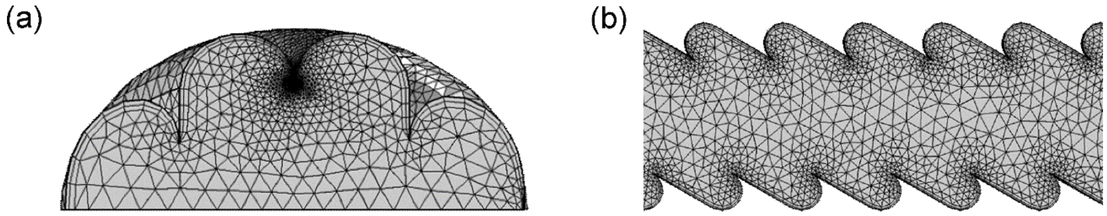

Fig. S2 Schematic of mesh of computation domain; (a) cross section of half channel and (b) cross section view cut along the channel length.

To simulate the flow in the mixing channel, the multi-physics module was combined with the laminar flow module for the velocity field; the transport of diluted species module for the concentration field was used at the stationary solver. In the laminar flow module, the Navier-Stokes equation with an assumption of steady and incompressible flow was used[1,2],

$$\rho(\mathbf{u} \cdot \nabla)\mathbf{u} = \nabla \cdot [-p\mathbf{I} + \mu(\nabla\mathbf{u} + (\nabla\mathbf{u})^T)] \quad (1)$$

where ρ is density, $\mathbf{u}$ is velocity vector, p is pressure, and μ is dynamic viscosity. The inlet velocity was calculated by dividing the cross-sectional area of the channel by the total flow rate, and the outlet pressure was used as the atmospheric pressure. For the transport of diluted species module, the advection-diffusion equation with an assumption of steady and incompressible flow

was used[3],

$$\nabla \cdot (-D_i \nabla c_i) + \mathbf{u} \cdot \nabla c_i = 0 \tag{2}$$

where c and D are the concentration and the diffusion coefficient, respectively. The inlet concentration was distributed through the step function with $1\times e^{-4}$ of the smooth zone between 1 (red color) and -1 (blue color), and a diffusion coefficient of 2.02×10^{-9} m^2/s, which is theoretically calculated and experimental value of water at 20ºC[4–6]. To minimize the overall calculation time, Eq. (1) was first calculated followed by solving Eq. (2). For the discretization order of each variable, a linear element and second order element were used for the pressure and velocity, respectively, considering a small change in pressure between inlet and outlet. The concentration of fluid was determined using a third order element for convergence Eq. (2). The Galerkin finite element method was utilized as a numerical scheme for consistent stabilization of streamline and crosswind diffusions[7–10].

Table S1 Detailed information regarding mesh settings.

**Channel design**	**90º-300 µm**			**30º-300 µm**		
**Mesh size**	**Coarser**	**Coarse**	**Normal**	**Coarser**	**Coarse**	**Normal**
**Number of elements**	495,486	1,226,542	2,155,191	394,184	951,463	2,504,097
**Max element size (mm)**	0.352	0.229	0.176	0.413	0.268	0.207
**Min element size (mm)**	0.088	0.0704	0.0528	0.103	0.0826	0.062
**Max element growth rate**	1.3	1.25	1.2	1.3	1.25	1.2
**Curvature factor**	0.9	0.8	0.7	0.9	0.8	0.7
**Resolution of narrow regions**	0.4	0.5	0.6	0.4	0.5	0.6
**Inlet velocity (mm/s)**	7.1996	7.1996	7.1996	6.0528	6.0528	6.0528

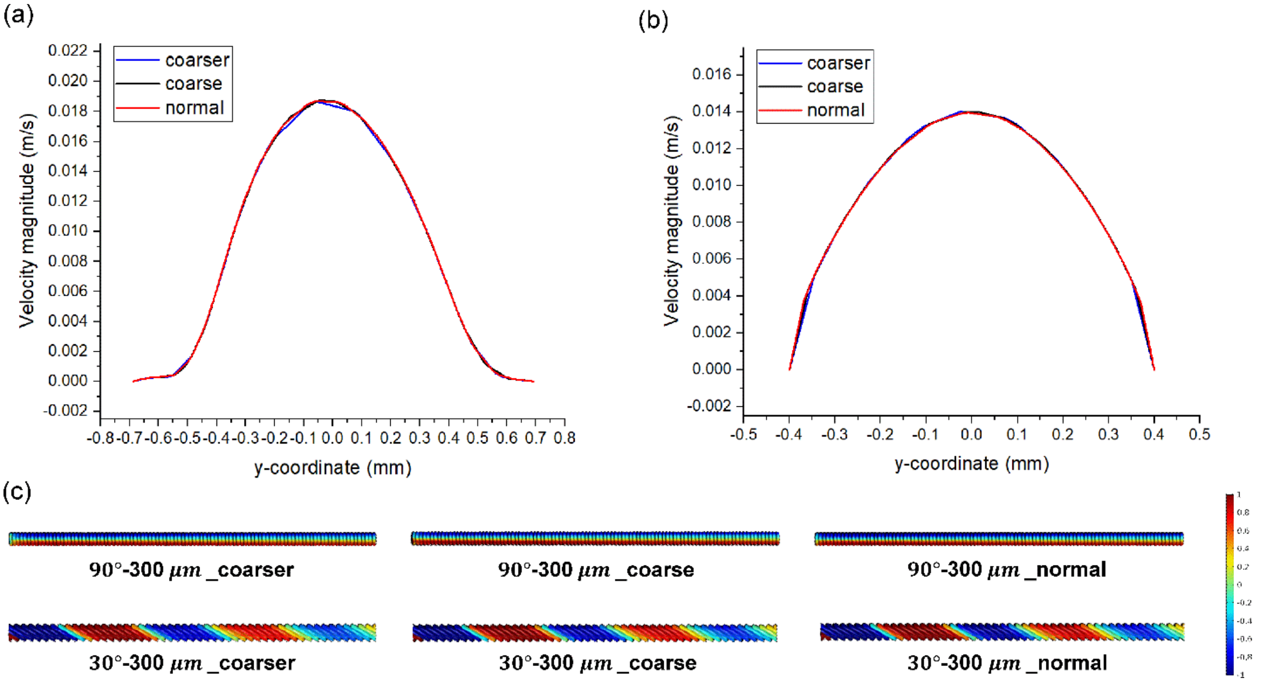

Fig. S3 Mesh independence study: velocity profile at center location of (a) “30º-300 μm”, (b) “90º-300 μm” channels, and (c) concentration of each channel.

The mesh (grid) independence test was also investigated for the accuracy of the simulation results, as shown in Fig. S3. The mesh independence test was conducted using “30º-300 μm” and the “90º-300 μm” channels. In the case of the mesh type, for both channels, the inside of the entire channel is composed of the tetrahedral shape, and the two-layer prism type mesh for the boundary layer is designed near the channel wall. Accordingly, the composition ratio of the mesh shape in the coarse mesh is tetrahedral of 85%, prisms of 13.8%, and pyramids of 1.2%, as shown in Figs. S2(a) and S2(b). More detailed information regarding the mesh type is listed in Table S1. The mesh independence studies were conducted using three different mesh sizes (coarser, coarse, normal) for both channels, and compared the values of velocity, pressure and concentration simultaneously. First, the pressure difference between the inlet and outlet of each channel was compared. In the “30º-300 μm” channel, values of the pressure difference were

12.391, 12.342, and 12.317 Pa for coarser, coarse, and normal mesh types, respectively. In the "90º-300 μm" channel, the pressure difference between the inlet and outlet was 9.6158, 9.5316, and 9.4788 Pa for coarser, coarse, and normal mesh types, respectively. When the values calculated from coarser and normal size meshes are compared, the error of the pressure difference in both channels is less than 1.5%. Regarding the velocity magnitude, values at the center of the "30º-300 μm" were 18.69, 18.75, and 18.68 mm/s for coarser, coarse, and normal mesh types, respectively (Fig. S3(a)). For the "90º-300 μm" channel, the velocities of 14.04, 14.02, and 13.97 mm/s were calculated using coarser, coarse, and normal mesh types, respectively, as shown in Fig. S3(b). Therefore, it was confirmed that computed flow behaviors were not significantly affected by the type of mesh.

To investigate the mesh independence on mixing concentration, the value of relative variance (S) derived from the flowing equation was used for quantitative comparison[11],

$$V_x = \int_{K_x} (c - c_0)^2 dA \,/\, \int_{K_x} dA \;,\; S = \frac{V_{outlet}}{V_{inlet}} \;\; (0 \le S \le 1) \qquad (3)$$

where V_x is the variance of concentration at inlet and outlet, c is concentration, and c_0 is average of concentration. As the value of S close to 0, it means that the two fluids are well mixed. The "30º-300 μm" channel showed the S of 0.076701, 0.084795, and 0.092019 for coarser, coarse, and normal meshes, respectively. The "90º-300 μm" channel exhibited the S of 0.54193, 0.57246, and 0.60186 for coarser, coarse, and normal meshes, respectively. Although there was a slight difference in the S with respect to the mesh type, the qualitative comparison exhibited no significant difference, as shown in Fig. S3(c), which was enough to evaluate the mixing performance between all designed channels. Based on this mesh independence study, all mixing channels were simulated using a coarse-sized mesh to estimate the flow trend efficiently and rapidly in this study.

II. CFD simulation: results

The simulation results are shown in Fig. S4. It was confirmed that the simulation showed the same tendency as the colorimetric experiments. In the 90º channel, the concentration distribution appeared parallel to the flow direction, whereas, in other channels, it was indicated that the concentration is distributed in the direction of the channel length owing to the flow along the surface raster pattern. In particular, at the channel cross-sectional flow in the 60º and 30º channels, it can be seen that one fluid surrounds the other, and this flow is repeated as the positions of the two fluids change. This flow is repeated faster as the surface raster angle is small and the pattern thickness increases which shows the same result as in the colorimetric experiment.

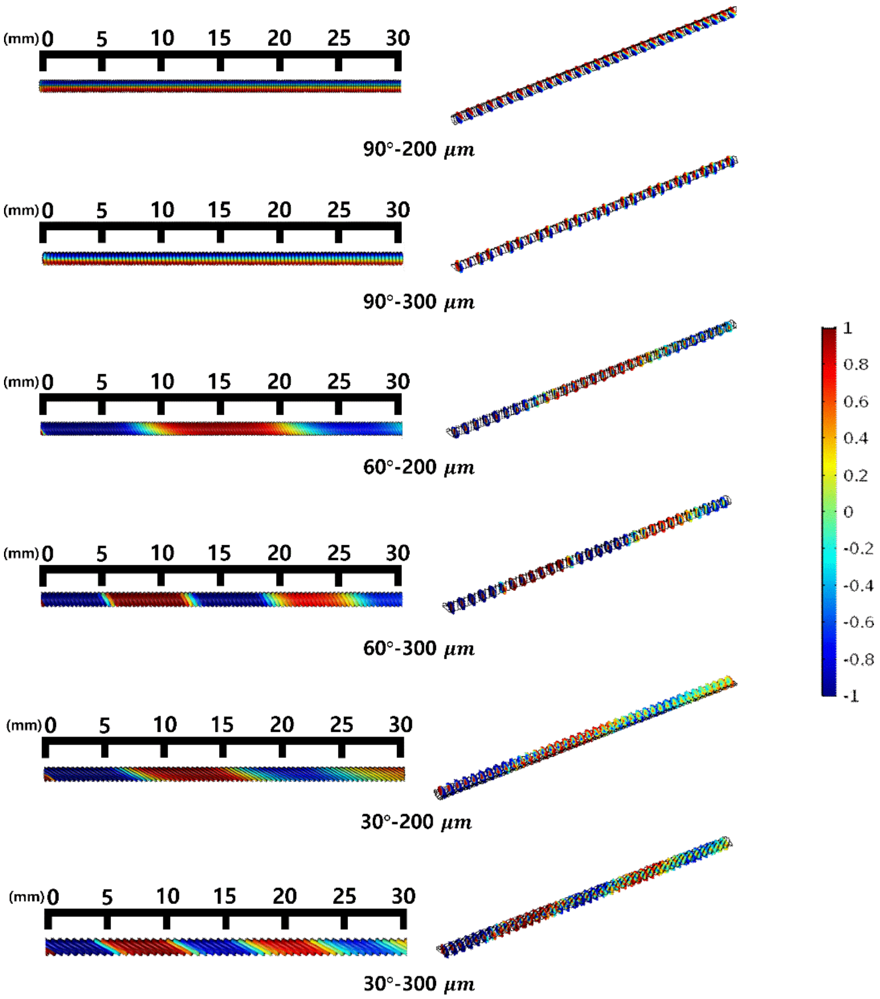

Fig. S4 The contour of concentration for each channel design (total flow rate = 240 μL/min).

Fig. S5 shows the simulation results and the colorimetric experiment results with respect to the total flow rate. The flow rate did not significantly affect the flow change, unlike the raster angle and pattern thickness of the surface at Reynolds (*Re*) numbers between 1 and 10. However, when the *Re* number exceeded 10, the speed at which the two fluids were reversed slightly increased, which is a different result from Eq. (15). This might be a phenomenon caused by the effect of friction related to the Darcy friction factor; the Darcy friction factor decreases linearly from a *Re* number of 10 or higher such that the fluid can flow with less friction[12].

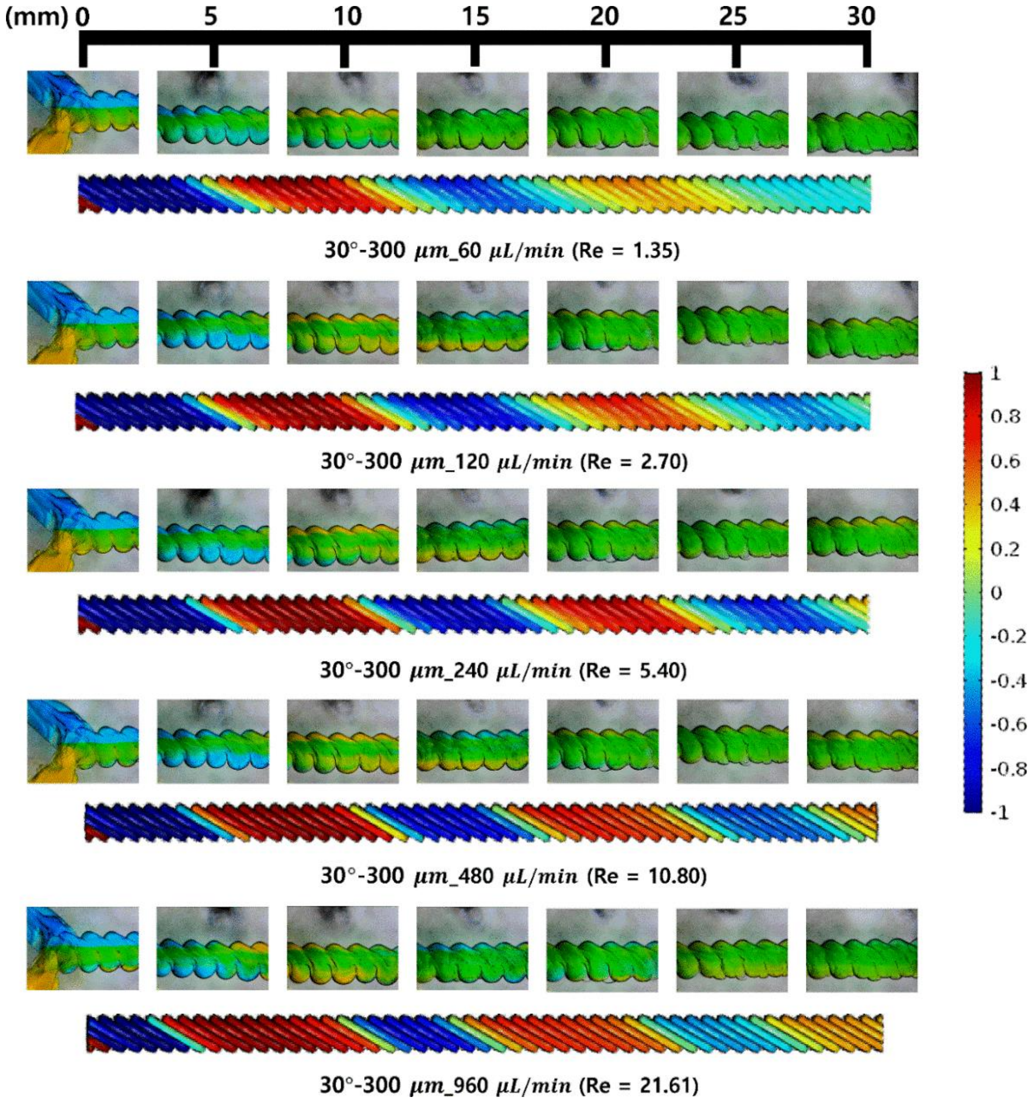

Fig. S5 Comparison of concentration between experiment and simulation results of "30º-300 μm" channel with various *Re* numbers.

REFERENCE

1. White, F. M. *Fluid Mechanics*, *8th edition*. (McGraw Hill, 2016).
2. Hirsch, C. *Numerical Computation of Internal and External Flows: The Fundamental of Computational Fluid Dynamics*, *2nd edition*. (BH, 2007).
3. Stocker, T. *Introduction to Climate Modeling, Corrected Version*. (Springer, 2014).
4. Holz, M., Heil, S. R. & Sacco, A. Temperature-dependent self-diffusion coefficients of water and six selected molecular liquids for calibration in accurate 1H NMR PFG measurements. *Phys. Chem. Chem. Phys.* **2**, 4740–4742 (2000).
5. Tofts, P. S. *et al.* Test liquids for quantitative MRI measurements of self-diffusion coefficient in vivo. *Magn. Reson. Med.* **43**, 368–374 (2000).
6. Easteal, A. J., Price, W. E. & Woolf, L. A. Diaphragm cell for high-temperature diffusion measurements. Tracer Diffusion coefficients for water to 363 K. *J. Chem. Soc. Faraday Trans. 1 Phys. Chem. Condens. Phases* **85**, 1091-1097 (1989).
7. Hauke, G. & Hughes, T. J. R. A unified approach to compressible and incompressible flows. *Comput. Methods Appl. Mech. Eng.* **113**, 389–395 (1994).
8. Brooks, A. N. & Hughes, T. J. R. Streamline upwind/Petrov-Galerkin formulations for convection dominated flows with particular emphasis on the incompressible Navier-Stokes equations. *Comput. Methods Appl. Mech. Eng.* **32**, 199–259 (1982).
9. Harari, I. & Hughes, T. J. R. What are C and h?: Inequalities for the analysis and design of finite element methods. *Comput. Methods Appl. Mech. Eng.* **97**, 157–192 (1992).
10. Bazilevs, Y., Calo, V. M., Tezduyar, T. E. & Hughes, T. J. R. YZβ discontinuity capturing for advection-dominated processes with application to arterial drug delivery. *Int. J. Numer. Methods Fluids* **54**, 593–608 (2007).
11. Cosentino, A. *et al.* An efficient planar accordion-shaped micromixer: From biochemical mixing to biological application. *Sci. Rep.* **5**, 1–10 (2015).
12. McKeon, B. J., Swanson, C. J., Zagarola, M. V., Donnelly, R. J. & Smits, A. J. Friction factors for smooth pipe flow. *J. Fluid Mech.* **511**, 41–44 (2004).
